# Carbohydrate antigen 125 on epicardial fat and its association with local inflammation and fibrosis-related markers

**DOI:** 10.1186/s12967-024-05351-z

**Published:** 2024-07-03

**Authors:** Sonia Eiras, Rafael de la Espriella, Xiaoran Fu, Diego Iglesias-Álvarez, Rumeysa Basdas, J. R. Núñez-Caamaño, J. M. Martínez-Cereijo, L. Reija, A. L. Fernández, David Sánchez-López, Gema Miñana, Julio Núñez, José R. González-Juanatey

**Affiliations:** 1 Translational Cardiology Group, Health Research Institute, Santiago de Compostela, Spain; 2 Centro de Investigación Biomédica en Red en Enfermedades Cardiovasculares (CIBERCV), Madrid, Spain; 3 Department of Cardiology, Hospital Clínico Universitario de Valencia (INCLIVA), Avda. Blasco Ibáñez 17, 46010 Valencia, Spain; 4 Coronary Unit. Cardiovascular Department, University Hospital of Santiago de Compostela, Santiago de Compostela, Spain; 5 Cardiology Group, Health Research Institute, Choupana, S/N, 15706 Santiago de Compostela, Spain; 6 Heart Surgery Department, University Hospital of Santiago de Compostela, Santiago de Compostela, Spain; 7 Department of Medicine, Universitat de València, Valencia, Spain; 8 University of Santiago de Compostela, Santiago de Compostela, Spain

**Keywords:** MUC16, CA125, Epicardium, Heart failure

## Abstract

**Background:**

Carbohydrate antigen 125 (CA125) is a proteolytic fragment of MUC-16 that is increased in heart failure (HF) and associated with inflammation, fluid overload, and worse adverse events. Our main objective was to study the expression of CA125 on epicardium and its association with inflammation, adipogenesis, and fibrosis.

**Methods:**

Epicardial fat biopsies and blood were obtained from 151 non-selected patients undergoing open heart surgery. Immunohistochemistry, ELISA, or real-time PCR were used for analyzing protein or mRNA expression levels of CA125 and markers of inflammatory cells, fibroblasts, and adipocytes. Epithelial or stromal cells from epicardium were isolated and cultured to identify CA125 and its association with the adipogenesis and fibrosis pathways, respectively.

**Results:**

The median age was 71 (63–74) years, 106 patients (70%) were male, and 62 (41%) had an established diagnosis of HF before surgery. The slice of epicardial fat biopsy determined a positive and colorimetric staining on the epithelial layer after incubating with the CA125 M11 antibody, providing the first description of CA125 expression in the human epicardium. Epicardial CA125 showed a strong and positive correlation with markers of inflammation and fibrosis in the epicardial fat tissue while exhibiting a negative correlation with markers of the adipogenesis pathway. This relationship remained significant after adjusting for potential confounders such as a prior HF diagnosis and plasma CA125 levels.

**Conclusion:**

Epicardial cells express CA125, which is positively associated with inflammatory and fibroblast markers in epicardial adipose tissue. These results suggest that CA125 may be biologically involved in HF progression (transition from adipogenesis to fibrosis).

**Graphical Abstract:**

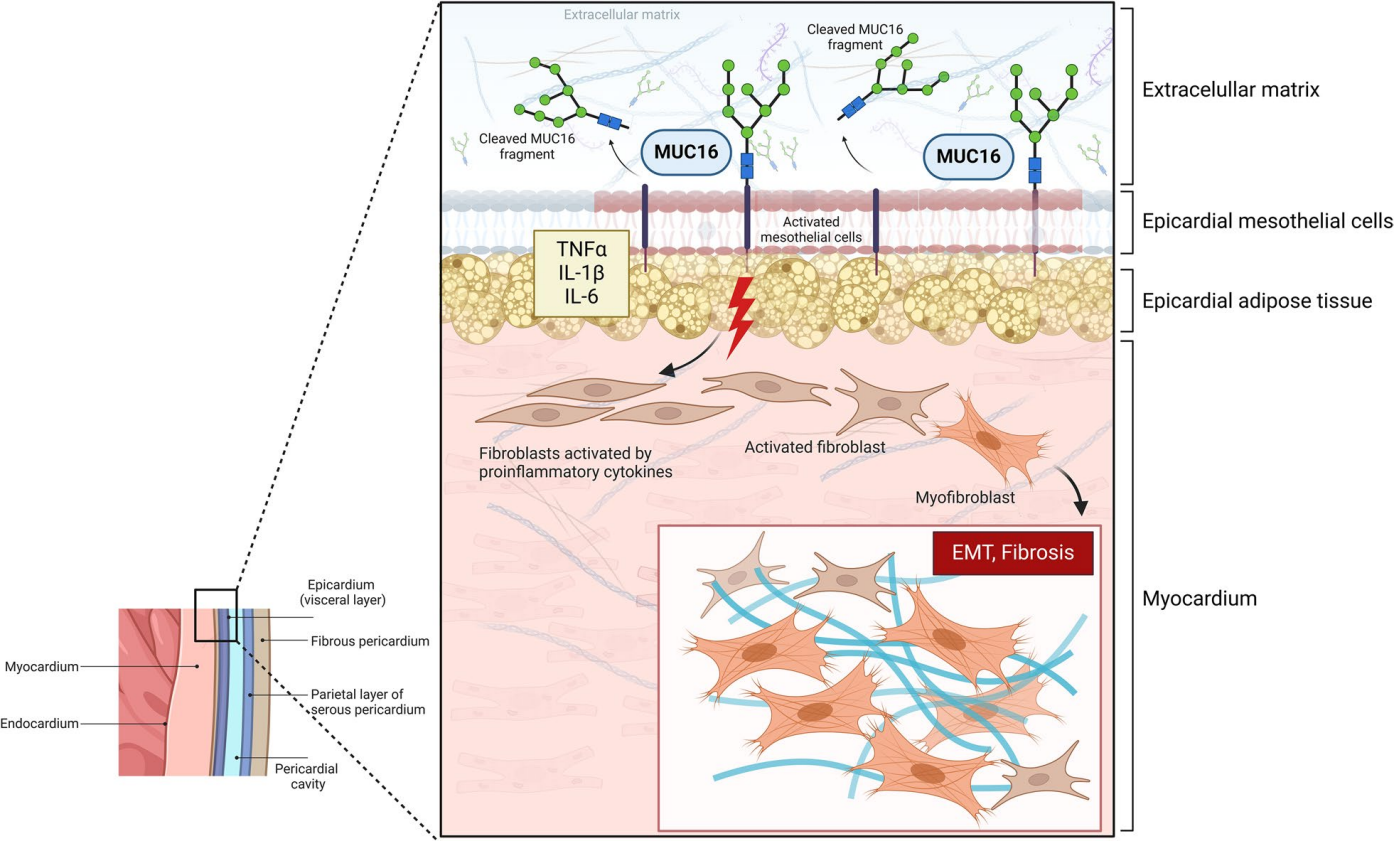

**Supplementary Information:**

The online version contains supplementary material available at 10.1186/s12967-024-05351-z.

## Highlights

- This is the first time that CA125 was identified on human epicardium biopsies and primary culture of epithelial cells was performed. This is a promising tool for understanding the pathophysiological mechanisms association between CA-125 and HF progression.

**Supplementary Information:**

The online version contains supplementary material available at 10.1186/s12967-024-05351-z.

## Introduction

Mucin 16 (MUC16), also known as cancer antigen 125 or carbohydrate antigen 125 (CA125), is a large (> 2200 amino acids) and heavily O-glycosylated transmembrane mucin that constitutes the glycocalyx of the epithelium and plays a crucial role in cellular interactions and signalling [1]. One proposed function of MUC16 is to protect epithelial surfaces from mechanical stress by acting as a lubricant [2]. However, it is also involved in various processes (physiological and pathological) such as fluid and cell transport, tissue repair, tumor dissemination, and modulating the immune response (including adhesive and anti-adhesive properties) [3].

Although CA125 has been extensively studied as a circulating biomarker for monitoring epithelial ovarian cancer, elevated plasma levels of CA125 are also observed in other malignancies and non-cancerous conditions such as heart failure (HF) [4]. In cancer, MUC16 overexpression is thought to result from altered gene responsiveness to factors in the tumour microenvironment and has been linked to attenuated cellular apoptosis, chemotherapy resistance, tumor growth, and metastasis [1]. In patients with HF, MUC16 upregulation and extracellular proteolytic cleavage appear to be related to biomechanical stress and inflammation [3, 4]. However, while substantial evidence supports the utility of CA125 for risk stratification, monitoring, and guiding HF depletive therapy [4, 5], it remains unclear whether this glycoprotein merely serves as a surrogate marker of fluid overload and inflammation or plays an active role in the pathobiology of the disease.

The human epicardium is characterized histologically by loose connective tissue containing autonomic nerves and variable amounts of adipose tissue [6]. Additionally, it is covered by a simple mesothelium that also lines the pericardial space [6]. Under normal conditions, the epicardial adipose tissue (EAT) is metabolically active and performs beneficial paracrine and exocrine functions [7]. However, in the presence of biomechanical stress (e.g., HF, valvular disease) and inflammatory disorders (e.g., diabetes mellitus, obesity, coronary artery disease), EAT shifts its biology to a pro-inflammatory state, becoming a source of several pro-inflammatory cytokines (leptin, tumor necrosis factor-alpha, interleukin (IL)-1B and IL-6) [8, 9]. These epicardial-released adipokines have been associated with fibroblast proliferation, collagen synthesis, and myofibroblast activation [10]. Given that MUC16 can potentially be expressed in all mesothelial cells (including epicardium) and it is known to participate in modulating key fibrotic cellular processes such as epithelial-mesenchymal transition (EMT), fibroblast to myofibroblast transition, and fibroblast proliferation [1, 11], we speculate that epicardial MUC16 could be associated with inflammatory and fibrotic processes in the human heart.

This study aims to explore (1) whether MUC16 is expressed in the epicardial layer of the human heart; and (2) to analyze the interplay between MUC16 epicardial expression and plasmatic levels with adipogenesis, inflammatory, and fibrosis pathways at the tissue level.

## Methods

### Patients

A total of 151 non-selected patients who underwent open heart surgery because of severe valvular heart disease and/or coronary artery disease between 2020 and 2023 were enrolled. Patients with prior heart surgery and severe infection were excluded. The decision to exclude patients with severe infections was based primarily on the clinical judgment of the treating physicians, relevant laboratory findings, and results from antibacterial cultures. The study complied with the Declaration of Helsinki and was approved by the Galician Clinical Research Ethics Committee (protocol code 2019/439 and 2023/240), and written informed consent was obtained from all participants. The diagnosis of HF was obtained by reviewing individual patients’ medical records and was defined as an established diagnosis of HF requiring at least intermittent need for oral diuretic treatment or having either made an urgent visit to the emergency department or been hospitalized for HF within 12 months prior to heart surgery.

### Biopsies and blood samples

Epicardial (EAT) and subcutaneous fat (SAT) (0.2–0.5 g) segments were collected from the atrioventricular/interventricular groove of the heart and thoracic region, respectively, during surgery and immediately transferred to the laboratory on ice. Fat biopsies were used for cell culture isolation or stored at -80 ºC for further RNA extraction. Blood samples were collected before surgery (on the same day) and centrifuged at 1800x*g *for 10 min. Subsequently, plasma was stored at − 80 °C until used.

### Immunohistochemistry

Epicardial fat biopsies were fixed in 10% neutral buffered formalin for 24 h and then embedded in paraffin. Sections 4 µm-thick were mounted on FLEX IHC microscope slides (Agilent, Carpinteria, CA). The sections underwent deparaffination, followed by epitope retrieval. Immunohistochemistry was then performed automatically using an AutostainerLink 48 immunostainer (Agilent). Briefly, the process included the following steps: the slides were incubated at room temperature with anti-CA125 M11 antibody (Ready to use, Agilent Dako Omnis, Santa Clara, USA). This was followed by incubation with anti-mouse immunoglobulins conjugated with peroxidase-labelled dextran polymer (EnVision +, Dako). The sections were then treated with DAB + substrate-chromogen solution and EnVision FLEX hematoxylin (Agilent), according to the manufacturer´s protocol.

### Epithelial mesothelial cell culture and CA125 immunofluorescence and flow cytometry

Under sterile conditions, the epithelial layer from epicardial fat biopsies was extracted, and cells were cultured for 15 days using a cell growth medium (Cell Applications Inc, San Diego, USA). Subsequently, the mesothelial cells were seeded in a 24-well plate (0.3 or 1 × 106 /well; NEST BioTechnologies Co., Wuxi, China), washed with saline solution, and fixated with 200ul of a 1:1 methanol-acetone (Merck, New Jersey, USA) for 15 min at room temperature (RT). Afterward, the cell membrane was permeabilized using Triton X-100 (0.1%) (Sigma-Aldrich, St Louis, MO, USA) for 5 min at RT and blocked with 1% bovine serum albumin (BSA) (Sigma-Aldrich) for 30 min at 37 °C. After washing with saline solution, cells were incubated overnight at 4 °C with CA125 M11 antibody. The following day, the cells were washed and incubated with anti-Mouse IgG Alexa Fluor 488 (1 µg/mL, Invitrogen, Life Technologies Corporation, Eugene, USA) at RT in the dark for 1 h. Lastly, cells were counterstained with NucBlue™ Fixed Cell ReadyProbes™ (DAPI; Invitrogen) and visualized with fluorescence microscope Axio Vert A1 (ZEISS).

Flow cytometry analysis was used to determine the percentage of CA125 positive cells in EAT biopsy and primary culture of mesothelial cells. Briefly, EAT biopsy, after being washed with phosphate-buffered saline (PBS, Gibco Life Technologies Limited, Paisley, UK) for 1 h at 4 ºC, was homogenized with a scalpel and mixed with M199 with Earle’s Salts (0.25 mL, Gibco) and collagenase type I (345 UI/mL, Gibco). After isolating the stromal cells with collagenase and tripsinized the primary culture of mesothelial cells from epicardium, cells were centrifuged at 400*g* for 5 min and resuspended in blocking buffer (5% BSA /TBS-Tween) for 30 min at 4 ºC in dark. After washing, cells were resuspended in the primary antibody against CA125 (IR70161-2; Dako, Agilent, Santa Clara, United States) for 30 min at 4 ºC. After the incubation, cells were washed with PBS and incubated with the secondary antibody anti-mouse Alexa Fluor 488 (200 μg/mL; Invitrogen, Life Technologies) for 30’ at room temperature. Finally, cells were fixated using 1% paraformaldehyde/PBS and measured with a BD FACSCanto™ II Clinical Flow Cytometer (BD Biosciences). Percentage of positive and negative cells were analyzed by FlowJo (BD Biosciences).

### CA125 plasma levels

Plasma CA125 was measured using a commercially available solid-phase sandwich ELISA (enzyme-linked immunosorbent assay) kit (Thermo Fisher Scientific, Waltham, MA, USA) for research use only (not for use in diagnostic procedures). The intra-assay precision (coefficient of variation) is < 10%, and the inter-assay precision (coefficient of variation) is < 12%, with an analytical range of 0.55–400 U/mL.

### RNA isolation and real time-polymerase chain reaction

Total RNA was extracted from adipose tissue samples using the Rneasy kit (Qiagen, Hilden, Germany), following the manufacturer´s protocols. The extracted RNA was then reverse transcribed into cDNA using the Maxima First Strand cDNA Synthesis Kit (Thermo Fisher Scientific, Waltham, MA, USA). For gene expression analysis, 2 μL of cDNA was amplified using FastStart SYBR Green Master (Roche Diagnostics S.L., Barcelona, Spain). Real-time PCR analysis was performed using QuantStudio 3 (Thermo Fisher Scientific, Waltham, MA, USA). The amplification profile included an initial step at 95 °C for 30 s, followed by 40 cycles at 60 °C for 60 s and 72 °C for 30 s. Specific primers were used for this process [12]. Adiposity markers, leptin and FABP4, mesothelial marker intelectin-1 (ITLN-1), neutrophil, monocytes, and fibroblasts-related markers were determined as previously described [12]. The primers used for CA125 amplification were as follows: forward-CAACCTCCCCCATT and reverse-associate-ATCTGAAGTGTGGCTCAGCT. The genes' cycle threshold (Ct) values were normalized by the Ct values of actin (ACTB). The differential expression was represented as the antilogarithm of the ratio of Ct values of the gene of interest to Ct values of ACTB.

### Adipogenesis assay

Adipogenesis was induced in stromal vascular cells derived from epicardial fat biopsies of 15 patients, following a previously established protocol [13]. The adipogenic cocktail, named IDMT, consisted of 5 µg/ml insulin, 250 nM dexamethasone (DEX), 0.5 mM methylisobutylxanthine (MIX), and 1 µM thiazolidinedione (TZD), and was supplemented with 10% fetal bovine serum (FBS). All pharmacological components were sourced from Sigma–Aldrich. The cells were cultured in an M199 medium enriched with the IDMT cocktail, which was refreshed three times weekly for a period of 15 days. After the induction period, RNA and protein were isolated by Allprep DNA/RNA/protein mini kit (Qiagen, Hilden, Germany) following the manufacturer´s protocol. mRNA expression levels of adiponectin (ADIPOQ), fatty binding protein 4 (FABP4), CA125, and the mesothelial marker intelectin-1 (ITLN-1) were determined by real time-PCR using SybrGreen (FastStart SYBR Green Master (Roche Diagnostics, Mannheim, Germany) and specific primers (CA125: designed primers from sequence (NM_024690.2); Forward: 5′-CAACCTCACCTCCTCCCATT-3′ and Reverse: 5′-ATCTGAAGTGTGGCTCAGCT-3′) and conditions previously described [12]. The Ct values for these genes were normalized against the Ct values of ACTB. Differential expression was represented as the antilogarithm of the Ct gene/Ct ACTB. To evaluate adipogenesis efficiency, a ratio was calculated between the mRNA expression levels in cells treated with the IDMT cocktail and those in control conditions (without the cocktail). This analysis was based on the observed increase in ADIPOQ and FABP4 levels following adipogenesis induction. In addition, FABP4 protein levels were confirmed by western blot analysis. Isolated proteins from the kit were separated in a 12% SDS-PAGE using vertical electrophoresis and following buffer (250 mM Tris-Base, 1.92 M Glycine, 0.5% SDS) at 40 mA for 120 min. Finally, separated proteins were transferred to fluorescence-adapted polyvinylidene difluoride (PVDF) membrane (Immobilon FL-Membrane, Merck Millipore) using the following buffer (25 mM Tris-Base, 192 mM glycine, 0.0125% SDS and 20% methanol) at 400 mA for 60 min. Afterward, membrane was washed, blocked with 5% bovine albumin serum for 60 min and incubated with primary mouse mAb anti-Actin and rabbit mAb anti-FABP4 (1 μg/mL; Invitrogen, Life Technologies Corp., Waltham, MA USA) and secondary antibody anti-mouse Alexa Fluor 488 (2 μg/mL) and anti-rabbit Alexa Fluor 532 (2 μg/mL; Invitrogen). Visualized proteins, as bands, were detected by fluorescence detector ChemiDoc MP (Bio-Rad) and quantified by ImageLab™ software (6.1.0 build 7 Standard Edition, Bio-Rad).

### Statistics

Continuous variables are expressed as mean (SD) or median (IQR), and categorical variables are presented as numbers (%). Baseline characteristics were compared between groups defined by the presence or absence of HF. For continuous variables, comparisons were made using the student’s t-test or the non-parametric Mann–Whitney U test where appropriate. Categorical variables were compared using the Chi-square test.

Spearman correlation was determined between epicardial/plasma CA125 and DEFA3, CXCR2, CD16, CD14, PREF1, COL1A2, FABP4, and CD36. The same sets of variables were included in hierarchical cluster analysis (agglomerative type) with the ward. The D2 linkage method and Spearman correlation matrix was used as dissimilarity measures.

The associations between plasma and epicardial CA125 levels and the fibrosis pathway (indicated by fibroblast markers: preadipocyte factor 1 [Pref-1] and collagen type I alpha 2 [COL1A2]), adipogenesis pathway (indicated by adipocyte markers: fatty acid-binding protein 4 [FABP4] and fatty acid translocase [CD36]), and inflammatory pathway (indicated by neutrophil markers: alpha defensin 3 [DEFA3] and chemokine receptor 2 [CXCR2], and monocyte markers: CD16 and CD14) were assessed using multivariate linear regression analysis. The linearity of all continuous covariates was individually tested, and the variable was transformed, if appropriate, with fractional polynomials. All estimates were adjusted for the diagnosis of HF, age, sex, and either plasma or epicardial CA125 levels.

Two-sided P values were statistically significant at less than 0.05. All analyses were performed using SPSS v25.0. (Software SPSS Inc.; Chicago, IL, USA) and Stata 15.1 (StataCorp, 2017, Stata Statistical Software: Release 15; StataCorp, LLC, College Station, TX). Dendrograms were implemented in R version 4.2.3 (R Foundation for Statistical Computing, Vienna, Austria).

## Results

The median age was 71 (63–74) years, 107 patients (71%) were male, and 62 (41%) had an established diagnosis of HF before surgery.

### CA125 expression on epithelial cells from epicardial fat biopsy

**Fig.1:**
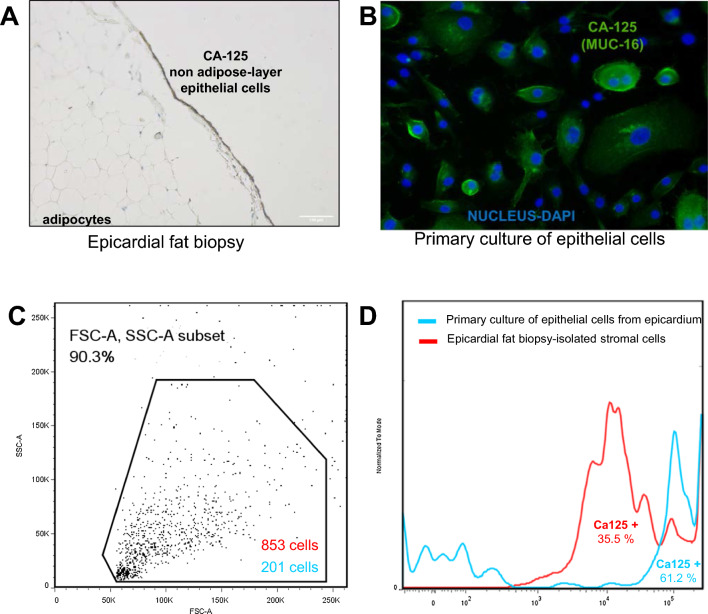
**A** Immunohistochemistry epicardial fat. CA125 staining on epithelial layer (brown). Microscope photograph 100×. Scale bar 100 μM. **B** Primary culture of epithelial layer from epicardial fat biopsy and CA125 staining (green) or nucleus staining (blue) with DAPI. Secondary antibody Alexa 488. Microscope photograph 200×. **C** SSC/FSC plot showing the gate used to sort epicardial fat biopsy-isolated stromal cells (total events = 853) or epithelial cells from epicardium primary culture (total events = 201) **D** Histogram representing the intensity of fluorescence by primary antibody CA125 and secondary antibody Alexa Fluor 488 and the percentage regarding total gated cells in epicardial fat biopsy-isolated stromal cells (35.5%) or epithelial cells from epicardium primary culture (61.2%). CA125, antigen carbohydrate 125

The slice of epicardial fat biopsy with epithelial, stroma, and adipose layer determined a positive and colorimetric staining on the epithelial layer after incubating with the CA125 M11 antibody (Fig. 1A). In addition, the culture of this layer, based on isolated mesothelial cells after mechanical disaggregation, has determined a viable primary culture with CA125 expression levels on the membrane surface after incubation with the antibody (Fig. 1B). Isolated stromal cells without epithelial layer were not stained with the antibody (data not shown). From the primary culture of epithelial cells derived from the epicardium, we successfully gated 201 cells and 61.2% were CA-125 positive. Additionally, from epicardial fat biopsy-isolated stromal cells, 853 cells were gated and 35.5% were CA-125 positive (Fig. 1C and D).

### Baseline characteristics according to the HF status

**Fig. 2:**
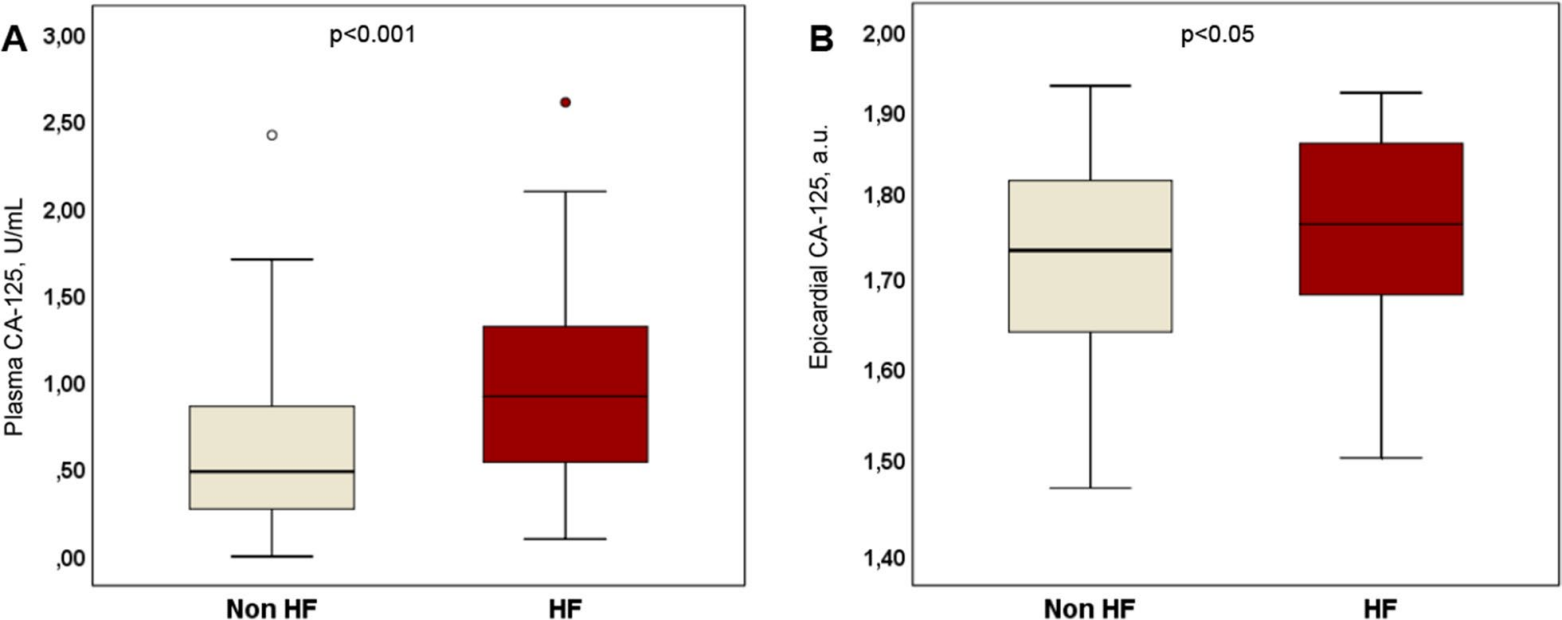
Box plot of **A** plasma and **B** epicardial (mRNA expression) CA125 levels from patients with and without heart failure (HF). CA125, antigen carbohydrate 125

The median of plasma and epicardial CA125 was 0.61 U/mL [interquartile range (IQR) 0.32, 1.1] and 1.74 (1.66, 1.83) a.u., respectively. The median plasma NT-proBNP (available in 95 patients) was 746 pg/mL (278, 1926). The baseline characteristics across HF status are summarized in Table 1. Overall, patients with a prior diagnosis of HF had lower LVEF and higher plasma NTproBNP, and were more frequently treated with beta-blockers, mineralocorticoid receptor antagonists, and renin-angiotensin system inhibitors). The indication for surgery was predominantly coronary artery disease or combined coronary and valvular. Plasma and epicardial CA125 values were higher in the population with an established diagnosis of HF (Fig. 2). The epicardial levels of inflammatory (DEFA3, CXCR2, CD16, CD14), fibrosis (PREF1, COL1A2), and adipogenesis (FABP4, CD36) biomarkers were similar across the HF status.

**Table 1:** Baseline characteristics across HF status before surgery

	No HF (n = 89)	HF (n = 62)	*p-value*
Demographics and medical history			
Age (years)	69 (61–73)	71 (64–74)	0.189
Male (n/%)	63 (71)	43(69)	0.850
Body mass index (kg/m^2^)	28 (25–31)	29 (25–32)	0.152
Hypertension (n/%)	59 (66)	49 (79)	0.088
Atrial fibrillation (n/%)	17 (21)	22 (39)	0.021
Diabetes (n/%)	22 (25)	20 (32)	0.309
Dyslipemia (n/%)	61 (68)	53 (85)	0.017
LVEF ≥ 50%	79 (98)	29 (51)	< 0.001
Indication for surgery (n/%)			0.552
Valvular	56 (65)	37 (60)	
Coronary	19 (22)	13 (21)	
Combined valvular and coronary	11 (13)	12 (19)	
Medications (n/%)			
Beta-blocker	45 (51)	44 (71)	0.012
ACEI/ARB	33 (45)	24 (54)	0.328
MRA	6 (7)	15 (24)	0.002
Laboratory values			
Hemoglobin, g/dl	13.7 (12.6–14.6)	14.0 (12.7–15.0)	0.431
Creatinine (mg/dl)	0.9 (0.8–1.0)	1.0 (0.8–1.3)	0.057
NT-proBNP (pg/ml)^a^	320 (139–839)	1323 (590–3149)	< 0.001
Plasma CA125 (U/ml)	0.5 (0.3–0.9)	0.9 (0.5–1.5)	< 0.001
Epicardial mRNA levels			
CA125 (a.u.)	1.7 (1.6–1.8)	1.8 (1.7–1.9)	0.047
Fibroblast markers			
PREF-1 (a.u.)	1.6 (1.5–1.6)	1.6 (1.6–1.7)	0.925
COL1A2 (a.u.)	1.9 (1.9–1.9)	1.9 (1.9–1.9)	0.572
Neutrophil markers			
DEFA3 (a.u.)	1.8 (1.7–1.9)	1.8 (1.7–1.9)	0.683
CXCR2 (a.u.)	1.7 (1.6–1.9)	1.7 (1.7–1.9)	0.991
Monocyte markers			
CD14 (a.u.)	1.8 (1.8–1.9)	1.8 (1.8–1.9)	0.741
CD16 (a.u.)	1.7 (1.6–1.7)	1.7 (1.6–1.8)	0.980
Adipocyte markers			
FABP4 (a.u.)	2.1 (2.1–2.2)	2.1 (2.1–2.2)	0.205
CD36 (a.u.)	2.0 (1.9–2.0)	1.9 (1.9–2.0)	0.133

Data are given as n (%) or median (IQR)

ACEI, angiotensin-converting enzyme inhibitor; ARB, angiotensin receptor blocker; CA125, carbohydrate antigen 125; CD36, fatty acid translocase; COL1A2, collagen type I alpha 2; CXCR2, chemokine receptor 2; DEFA3, alpha defensin 3; FABP4, fatty acid-binding protein 4; LVEF, left ventricular ejection fraction; MRA, mineralocorticoid receptor antagonists; NT-proBNP, N-terminal brain natriuretic peptide; PREF1, preadipocyte factor 1

^a^Data available in 101 patients

### Correlations and dendrogram

**Fig. 3:**
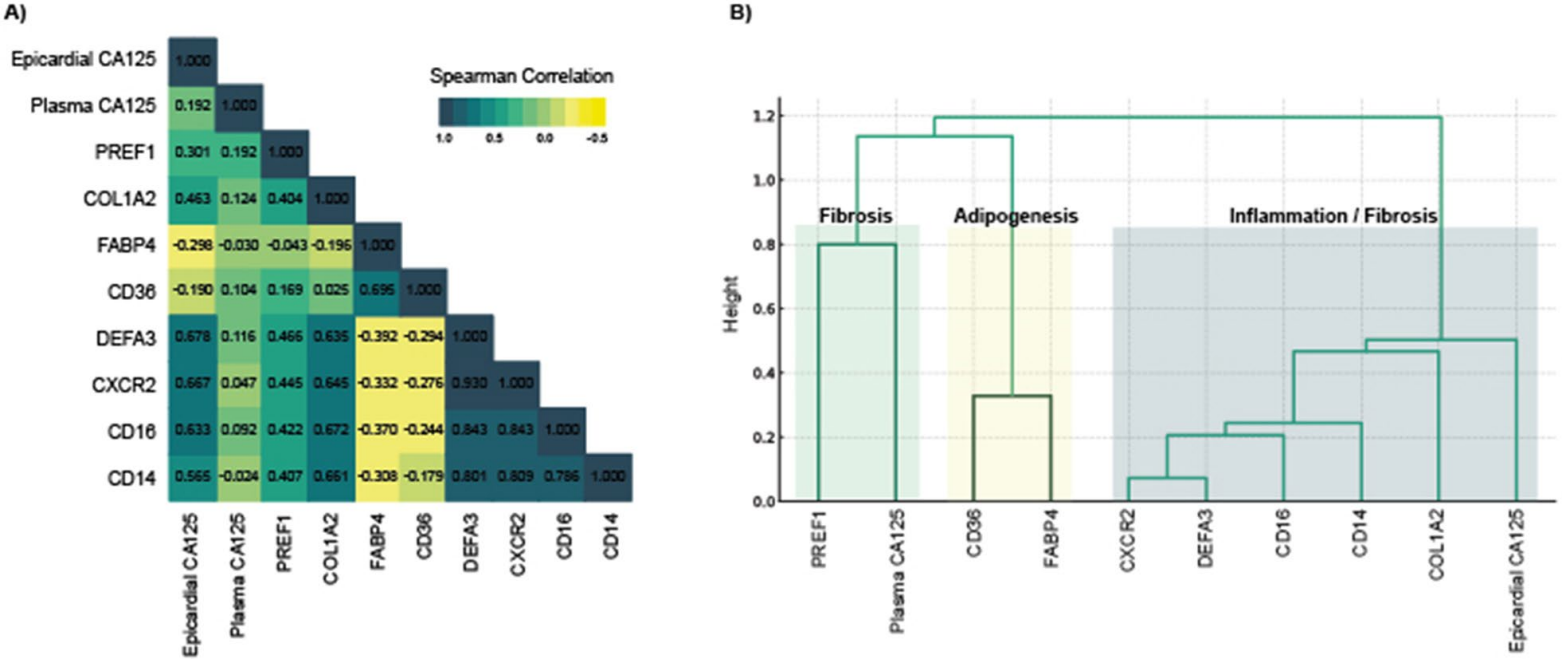
Heatmap and dendrogram. **A** Biomarker position of plasma and epicardial CA125 in a correlation heatmap. Correlations are based on Spearman’s rho as a correlation coefficient. **B** Biomarker position of plasma and epicardial CA125 in hierarchical cluster analysis. CA125, antigen carbohydrate 125; CD36, fatty acid translocase; COL1A2, collagen type I alpha 2; CXCR2, chemokine receptor 2; DEFA3, alpha defensin 3; FABP4, fatty acid-binding protein 4; PREF1, preadipocyte factor 1

Figure 3A displays the Spearman correlation heatmap coefficients of plasma and epicardial CA125 with epicardial inflammatory (DEFA3, CXCR2, CD16, CD14), fibrosis (PREF1, COL1A2), and adipogenesis (FABP4, CD36) biomarkers. Plasma and epicardial CA125 showed a positive weak correlation (rho 0.205; p = 0.011), which was not modified by the HF status (rho 0.193; p = 0.069 in patients with a prior diagnosis of HF vs. rho 0.147; p = 0.254 in those without the diagnosis of HF). Epicardial CA125 had the strongest and positive correlation with epicardial inflammatory (DEFA3 rho 0.678; p < 0.001, CXCR2 rho 0.667; p < 0.001, CD16 rho0.633; p < 0.001, CD14 rho 0.565; p < 0.001) and fibrosis markers (COL1A2 rho 0.463; p < 0.001). The correlation between epicardial CA125 and adipogenesis markers was inverse (FABP4 rho -0.298; p < 0.001, CD36 rho − 0.190; p = 0.025). Plasma CA125 showed a weak and non-significant correlation with DEFA3, CXCR2, CD16, CD14, FABP4, CD36, and COL1A2. The only positive (weak) and significant correlation was with epicardial PREF1 (rho 0.192; p = 0.024). The correlation between plasma CA125 and NTproBNP was positive and weak (rho 0.216; p = 0.028), whereas epicardial CA125 was not correlated with plasma NTproBNP (rho 0.090; p = 0.365).

In a hierarchical cluster analysis (Fig. 3B), epicardial CA125 clustered with the combined effect of fibrosis (COL1A2) and inflammatory (CD14, CD16, DEFA3, CXCR2) markers. Plasma CA125 clustered with PREF1.

### Plasma and epicardial CA125 levels and the fibrosis pathway

**Fig. 4:**
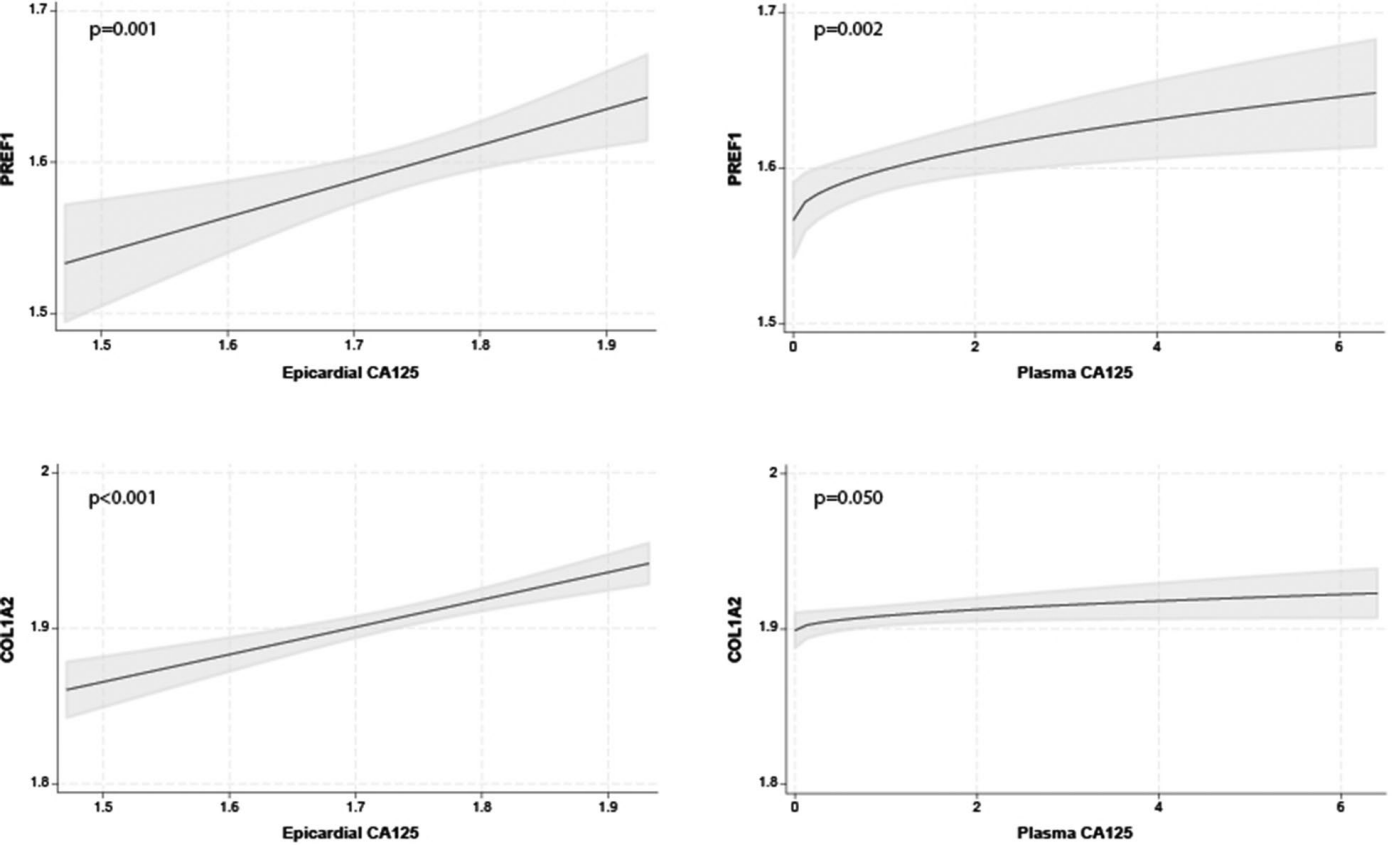
Relationship between epicardial (left) and plasma (right) CA125 and mRNA expression levels of fibroblasts markers (PREF1, COL1A2). CA125, antigen carbohydrate 125; COL1A2, collagen type I alpha 2; PREF1, preadipocyte factor 1

The multivariate regression analysis showed that the most important predictor of PREF1 and COL1A2 as fibrosis markers (line-up based on the magnitude of its contribution to the total R2 of the model) was epicardial CA125 (PREF1 ΔR2 = 0.075; p < 0.001, COL1A2 ΔR2 = 0.177; p < 0.001). The full model R2 was 0.177 and 0.208 for PREF1 and COL1A2, respectively. Plots depicting the relationship between the epicardial and plasma CA125 and fibrosis markers are shown in Fig. 4.

### Plasma and epicardial CA125 levels and the inflammatory pathway

**Fig. 5:**
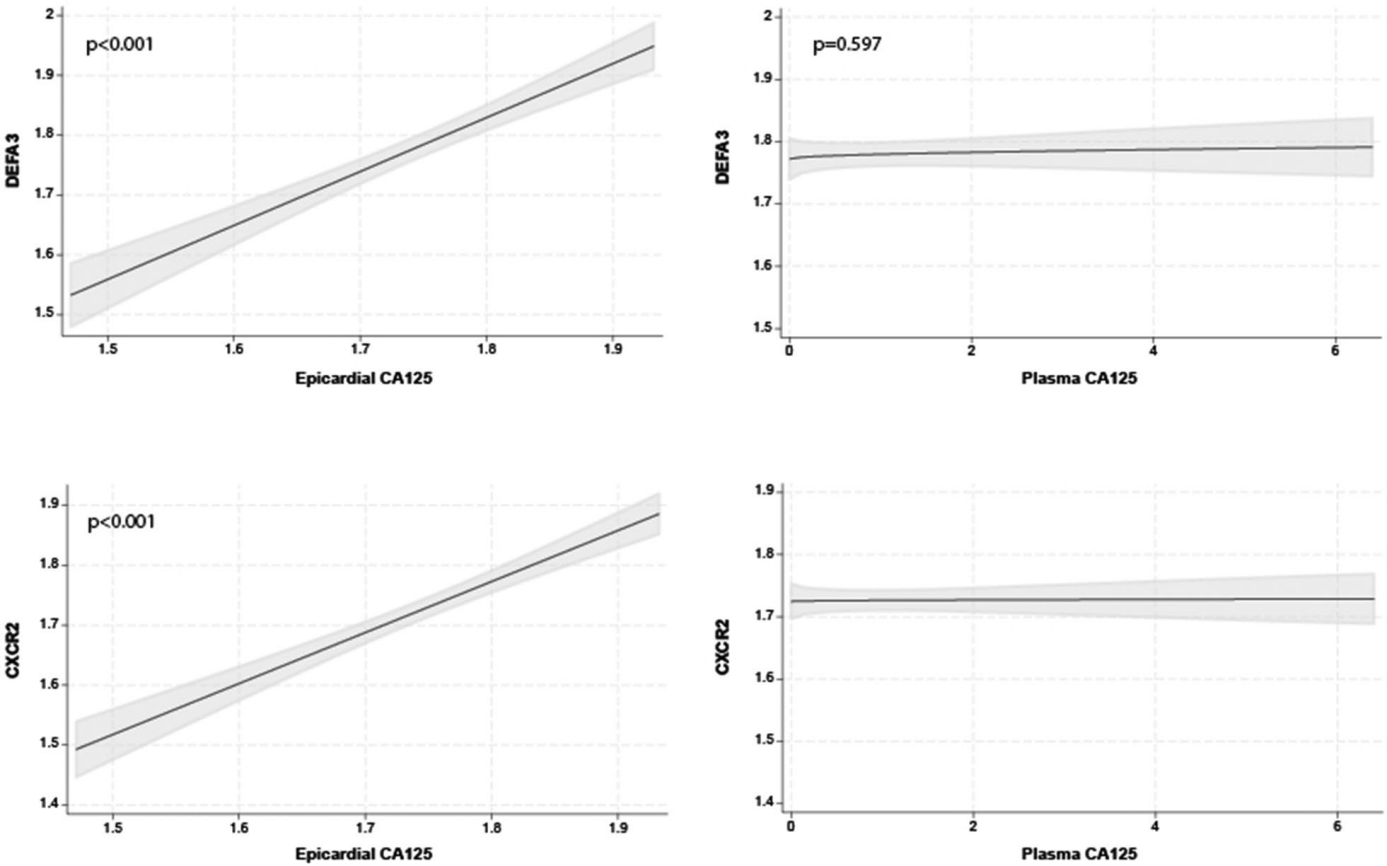
Relationship between epicardial (left) and plasma (right) CA125 and mRNA expression levels of neutrophils (CXCR2 and DEFA3). CA125, antigen carbohydrate 125; CXCR2, chemokine receptor 2; DEFA3, alpha defensin 3

**Fig. 6:**
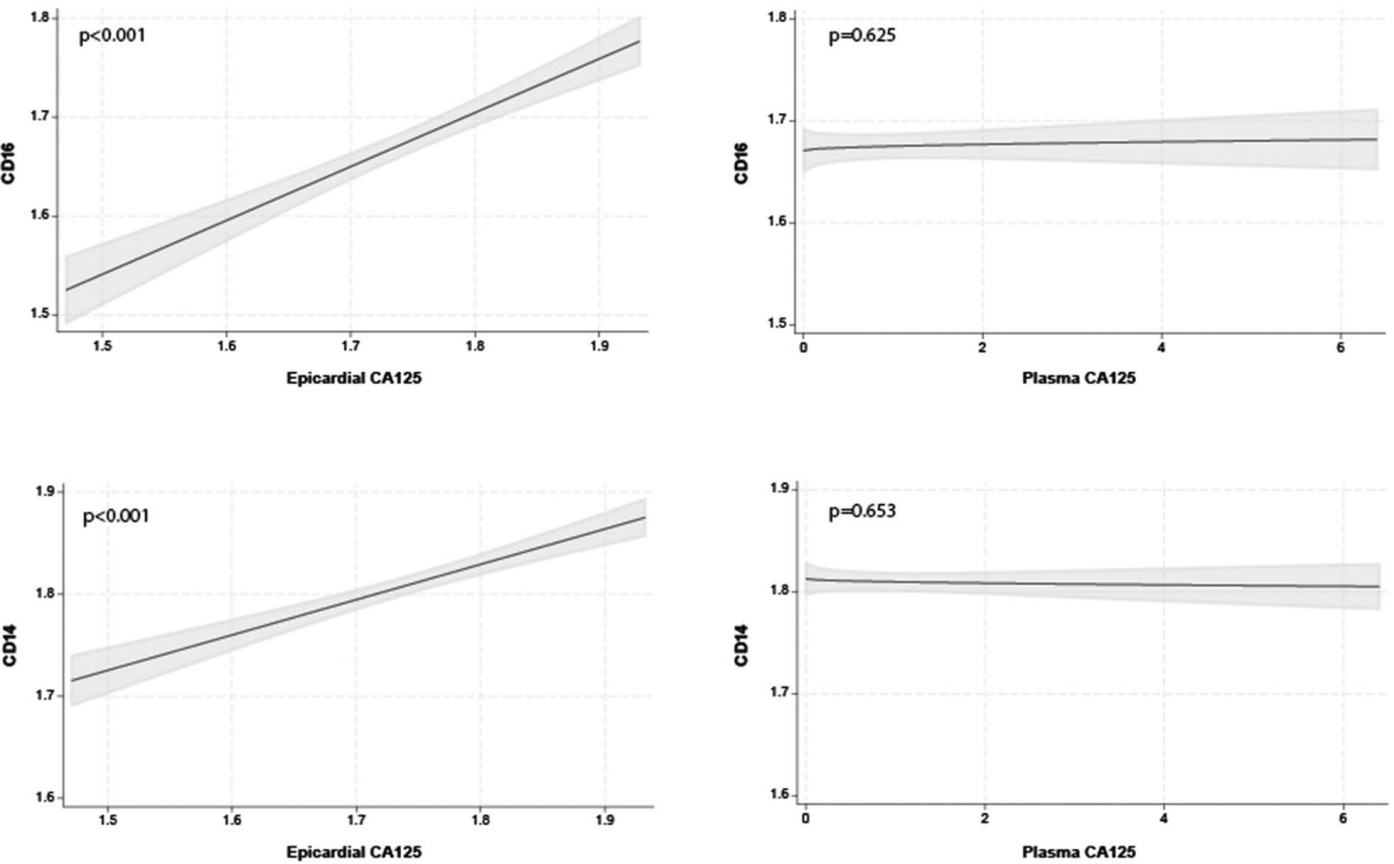
Relationship between epicardial (left) and plasma (right) CA125 and mRNA expression levels of monocytes (CD14, CD16). CA125, antigen carbohydrate

The multivariate regression analysis showed that the most important predictor of DEFA3 and CXCR2 as neutrophil markers (line-up based on the magnitude of its contribution to the total R2 of the model) was epicardial CA125 (ΔR2 = 0.399; p < 0.001, and ΔR2 = 0.443; p < 0.001). The full model R2 was 0.403 and 0.445 for DEFA3 and CXCR2, respectively. Plots depicting the relationship between the epicardial and plasma CA125 and neutrophil markers are shown in Fig. 5. Similarly, the most important predictor of CD16 and CD14 as monocyte markers (line-up based on the magnitude of its contribution to the total R2 of the model) was epicardial CA125 (ΔR2 = 0.385; p < 0.001, and ΔR2 = 0.330; p < 0.001). The full model R2 was 0.404 and 0.337 for CD16 and CD14, respectively. Plots depicting the relationship between the epicardial and plasma CA125 and neutrophil markers are shown in Fig. 6. Plasma CA125 was not associated with neutrophil or monocyte markers (Figs. 5 and 6).

### Plasma and epicardial CA125 levels and the adipogenesis pathway

**Fig. 7:**
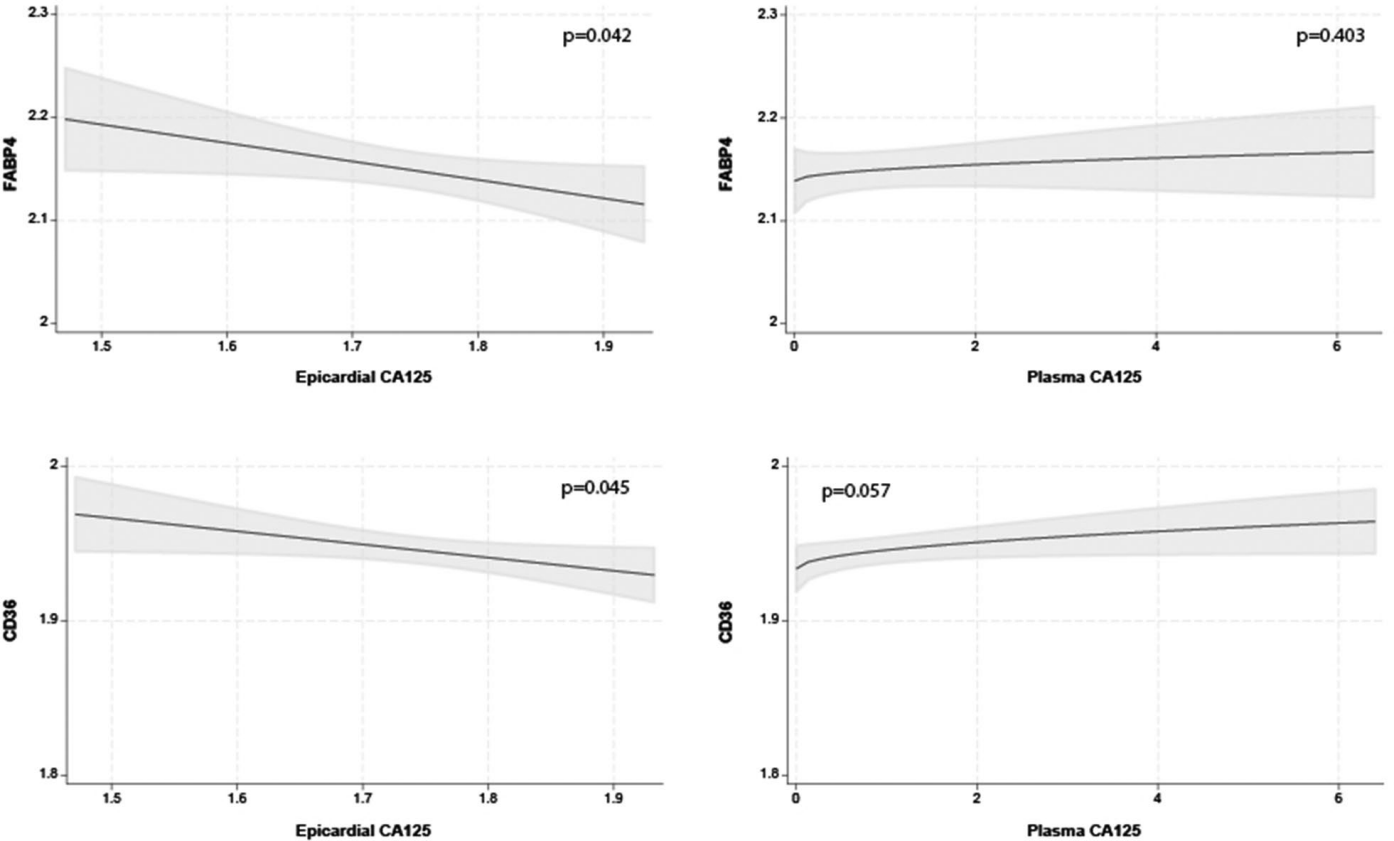
Relationship between epicardial (left) and plasma (right) CA125 and mRNA expression levels of adipocytes markers (FABP4 and CD36). CA125, antigen carbohydrate 125; CD36, fatty acid translocase; FABP4, fatty acid-binding protein 4

The multivariate regression analysis only marginally predicts FABP4 at the 5% level (R2 = 0.081; p = 0.039) and did not predict CD36 (R2 = 0.063; p = 0.060, respectively). Epicardial CA125 slightly improves the model but was not statistically significant (ΔR2 = 0.028; p = 0.080). The multivariate regression analysis showed that the overall model does not significantly predict CD36 at the 5% level (R2 = 0.063; p = 0.060). However, the direction of the association between epicardial CA125 and both markers was negative and linear (Fig. 7A and B). Plasma CA125 was not associated with FABP4 or CD36 (Fig. 7).

### CA125 expression levels on epicardial stromal cells and adipogenesis

Adipogenesis was induced in stromal vascular cells derived from epicardial fat biopsies of 15 patients. The median age was 67(63–76) years old, 13 patients (87%) were male, and the median BMI was 28 (26–32) kg/m^2^. A total of 8 (53%) patients had an established diagnosis of HF before surgery, and 8 had preserved LVEF (≥ 53%).

The expression of CA125 at basal conditions was positively related to ITLN-1 (R2 = 0.524; p = 0.002). However, after adipogenesis cocktail exposure, the presence of CA125 levels was positively associated with fibroblast marker PREF-1 (R2 = 0.280; p = 0.043) and negatively with adipogenesis induction, determined by adiponectin (ADIPOQ) (R2 = − 0.268; p = 0.035) and FABP4 increment (R2 = − 0.174; p = 0.031) (Supplementary Fig. 1).

## Discussion

The main findings of the present study are as follows: (i) This study provides the first description of CA125 expression in the epithelial layer of the human epicardium. Moreover, culturing this layer yielded a viable primary culture, demonstrating CA125 expression on the membrane surface following incubation with the M11 antibody; (ii) Plasma and epicardial CA125 showed a positive but weak correlation (non-statistically significant), suggesting a disconnection between CA125 expression in the epicardial layer and the plasmatic levels of its ectodomain, at least in stable clinical conditions; (iii) Intriguingly, and despite the weak correlation between plasma and epicardial CA125, patients with a prior diagnosis of HF had higher plasma and epicardial CA125 levels; (iv) Epicardial CA125 demonstrated a strong and positive correlation with markers of inflammation and fibrosis in the epicardial fat tissue while exhibiting a negative correlation with markers of the adipogenesis pathway. Furthermore, cluster analysis revealed that epicardial CA125 grouped with inflammation and fibrosis markers; (v) Multivariate adjustment confirmed a positive and statistically significant association between epicardial CA125 and markers of inflammation and fibrosis. The association between epicardial CA125 and adipogenesis markers was negative. Of note, the overall regression models were not effective in explaining a substantial portion of the variation in adipogenesis markers, highlighting the complexity of the underlying phenomena and suggesting the possible influence of unmeasured variables; and (vi) However, in a secondary analysis performed on 15 patients in whom adipogenesis was induced in stromal vascular cells derived from epicardial fat biopsies, the presence of epicardial mRNA CA125 levels was negatively correlated with the adipogenesis induction, after considering the levels of adipocytes markers, ADIPOQ and FABP4, regarding control. Contrary, a positive association was observed between epicardial CA125 and PREF-1 levels. Collectively, these findings underscore the putative role of epicardial CA125 overexpression on epicardial inflammatory and fibrosis pathways.

MUC16 is a large, membrane-associated mucin that contains 3 domains: N-terminal, tandem repeat, and C-terminal. The N-terminal and tandem repeat domains are highly glycosylated and extracellular, whereas the C-terminal domain contains multiple extracellular modules, a transmembrane domain, and a cytoplasmic tail^14^. MUC16 is known to be normally expressed in the peritoneum, pleura, pericardium, ocular epithelia, and the respiratory and female reproductive tracts [3, 4]. The present study adds to current knowledge by providing first-time evidence of MUC16 expression in the epicardium of the human heart and a first-time description of a viable primary culture demonstrating MUC16 or CA125 expression on the membrane surface following incubation with the M11 antibody.

Autoproteolytic cleavage, fibrotic factors, and neutrophil elastase are implicated in the enhanced shedding of MUC16 from the cell surface. After cleavage, the MUC16 C-terminal domain is known to translocate to the nucleus and bind to chromatin, thus potentially influencing gene expression regulation. Interestingly, the C-terminal domain has been demonstrated to be involved in MUC16-mediated oncogenic signaling and to induce fibro-proliferative disorders, whereas the N-terminal region has multiple sites for O-glycosylation, which may allow for extracellular matrix interactions [1].

### MUC16 levels and their association with inflammation and fibrosis

In HF, cell surface overexpression and release of MUC16 proteolytic fragments (detected by commercially available antibodies in plasma as CA125) appear to be related to congestion and inflammation [3, 4, 14, 15]. In this clinical context, plasma CA125 levels fluctuate with congestion status (particularly tissue congestion) during decompensation and clinical improvement, making it a useful circulating biomarker for risk stratification, monitoring congestion, and guiding therapy [16, 17]. However, CA125 is also involved in various processes, such as fluid and cell transport, inflammation, tissue repair, tumor dissemination, and immune response modulation [1].

In the present study, we found a strong and positive association between epicardial CA125 and inflammatory and fibroblast markers levels on epicardial fat. This relationship remained significant after adjusting for potential confounders such as a prior HF diagnosis and plasma CA125 levels. Moreover, given the observed disconnection between plasma and epicardial CA125, it is possible that local inflammatory and profibrotic mediators in the epicardial fat tissue might be influencing epicardial CA125 overexpression even in the absence of systemic congestion/inflammation [9, 10, 18]. Furthermore, epicardial CA125 core O- and N-glycans may interact with other extracellular matrix proteins like the family of galectins, which act as ligands between glycoproteins and glycolipids at the cell surface, potentially enhancing transmembrane signaling, with the subsequent activation of profibrotic transduction pathways [19–21]. Accordingly, we postulate that epicardial CA125 overexpression in HF (as demonstrated in the present study) might be involved in key fibrotic cellular processes.

### MUC16 levels and adipogenesis

Current evidence supports that CA125 plays an active role in promoting epithelial to mesenchymal transition, a biological process allowing polarized, immobile epithelial cells to undergo biochemical changes that enable them to assume a mesenchymal cell phenotype [1, 11]. This change results in increased migratory capacity, invasiveness, elevated resistance to apoptosis, and greatly enhanced production of extracellular matrix components [1]. Although we cannot confirm causality with the present findings, the inverse correlation between epicardial CA125 levels in stromal cells and the adipogenesis process may offer some preliminary insights into this hypothesis. Given the positive relationship between plasma and epicardial CA125 levels with fibroblast markers on epicardial fat, a putative explanation for the observed reduced adipogenesis in stromal cells at higher epicardial CA125 levels is an increased presence of fibroblasts following stromal isolation inhibiting adipogenesis. In fact, in the subgroup of patients in whom adipogenesis was induced in stromal vascular cells derived from epicardial fat biopsies, the presence of epicardial CA125 levels was negatively correlated with the adipogenesis marker ADIPOQ or FABP4 and positively correlated with PREF-1. However, further research is needed to comprehend the biological role of epicardial CA125 overexpression in patients with overt heart failure.

Several limitations need to be acknowledged. First, this is a single-center observational study, which, by design, can lead to residual (and unmeasured) confounding factors. Second, reliance on ICD codes and diuretic treatment for HF diagnosis without natriuretic peptide confirmation in most patients. Third, plasma CA125 was measured using a commercially available ELISA kit for research use only. Accordingly, we cannot corroborate the results with commercially available kits for use in diagnostic procedures. Fourth, the study was conducted in a population undergoing cardiac surgery, without HF being the main diagnosis. Thus, our results are merely hypothesis-generating in the HF field. Fifth, the adipogenesis assay was only performed in 15 patients. Therefore, it is not possible to extrapolate the results obtained to the rest of the study population. Finally, although our results only suggest an association (and not causality) between epicardial CA125 with inflammatory and fibrotic pathways in epicardial fat tissue, it has allowed the postulation of possible hypotheses that could provide rational explanations for the prognostic role of CA125 in HF.

## Conclusions

Epicardial cells express CA125, which is positively associated with inflammatory and fibroblast markers in epicardial adipose tissue. Primary culture of epicardial mesothelial cells might be a promising tool for understanding the pathophysiological association between CA125 and HF progression (transition from adipogenesis to fibrosis).

### Translational perspective

The identification of CA-125 in epicardial cells, its higher levels in heart failure and its association with local inflammatory and fibrosis markers suggest the possible role of this molecule on physiopathological mechanisms of HF progression.

### Supplementary Information

Additional file 1: Figure S1. Adipogenesis induction on epicardial stromal cells. **A** Relationship between mRNA expression levels of CA-125 and intelectin-1 (ITLN-1). **B** Protein levels of adipocyte marker, fatty acid binding protein 4 (FABP4) after or not adiponenesis induction (IDMT), analyzed by western blot (left) and quantified by Image J, density of bands was represented regarding actin (right). **C** Relationship between mRNA expression levels of CA-125 and adipogenesis induction (ratio between adipocyte marker ADIPOQ mRNA levels with *vs.* without IDMT. **D** Relationship between mRNA expression levels of CA-125 and fibroblast marker, PREF-1 in epicardial stromal cells after adipogenesis induction. **E** mRNA expression levels of CA-125 in adipogenesis-induced cells in patients with and without heart failure (HF). * and *** determined statistical significance between groups p < 0.05 and p < 0.001, respectively. ADIPOQ, adiponectin; CA125, antigen carbohydrate 125; INTL-1, intelectin-1.

## Data Availability

All data generated or analyzed during this study are included in this article. Further enquiries can be directed to the corresponding author.

## Abbreviations

CA125: Carbohydrate antigen 125
MUC-16: Mucin 16
HF: Heart failure
SEA: Sea urchin sperm protein, enterokinase, and agrin
EAT: Epicardial adipose tissue
SAT: Subcutaneous adipose tissue
FABP4: Fatty acid binding protein 4
DEFA3: Defensin alpha 3
CXCR2: C–X–C-motif chemokine receptor 2
PREF-1: Preadipocyte factor 1
COL1A2: Collagen type I alpha 2
CD36: Fatty acid translocase
ADIPOQ: Adiponectin
