# Supplementary material for: Carbohydrate antigen 125 on epicardial fat and its association with local inflammation and fibrosis-related markers
Source: J Transl Med. 2024 Jul 3;22:619. doi: 10.1186/s12967-024-05351-z (PMC11223376; doi:10.1186/s12967-024-05351-z)
Supplement: Supplementary file 1 — Additional file 1: Figure S1. Adipogenesis induction on epicardial stromal cells. A Relationship between mRNA expression levels of CA-125 and intelectin-1 (ITLN-1). B Protein levels of adipocyte marker, fatty acid binding protein 4 (FABP4) after or not adiponenesis induction (IDMT), analyzed by western blot (left) and quantified by Image J, density of bands was represented regarding actin (right). C Relationship between mRNA expression levels of CA-125 and adipogenesis induction (ratio between adipocyte marker ADIPOQ mRNA levels with vs. without IDMT. D Relationship between mRNA expression levels of CA-125 and fibroblast marker, PREF-1 in epicardial stromal cells after adipogenesis induction. E mRNA expression levels of CA-125 in adipogenesis-induced cells in patients with and without heart failure (HF). * and *** determined statistical significance between groups p < 0.05 and p < 0.001, respectively. ADIPOQ, adiponectin; CA125, antigen carbohydrate 125; INTL-1, intelectin-1. [file 12967_2024_5351_MOESM1_ESM.ppt]

## Slide 1
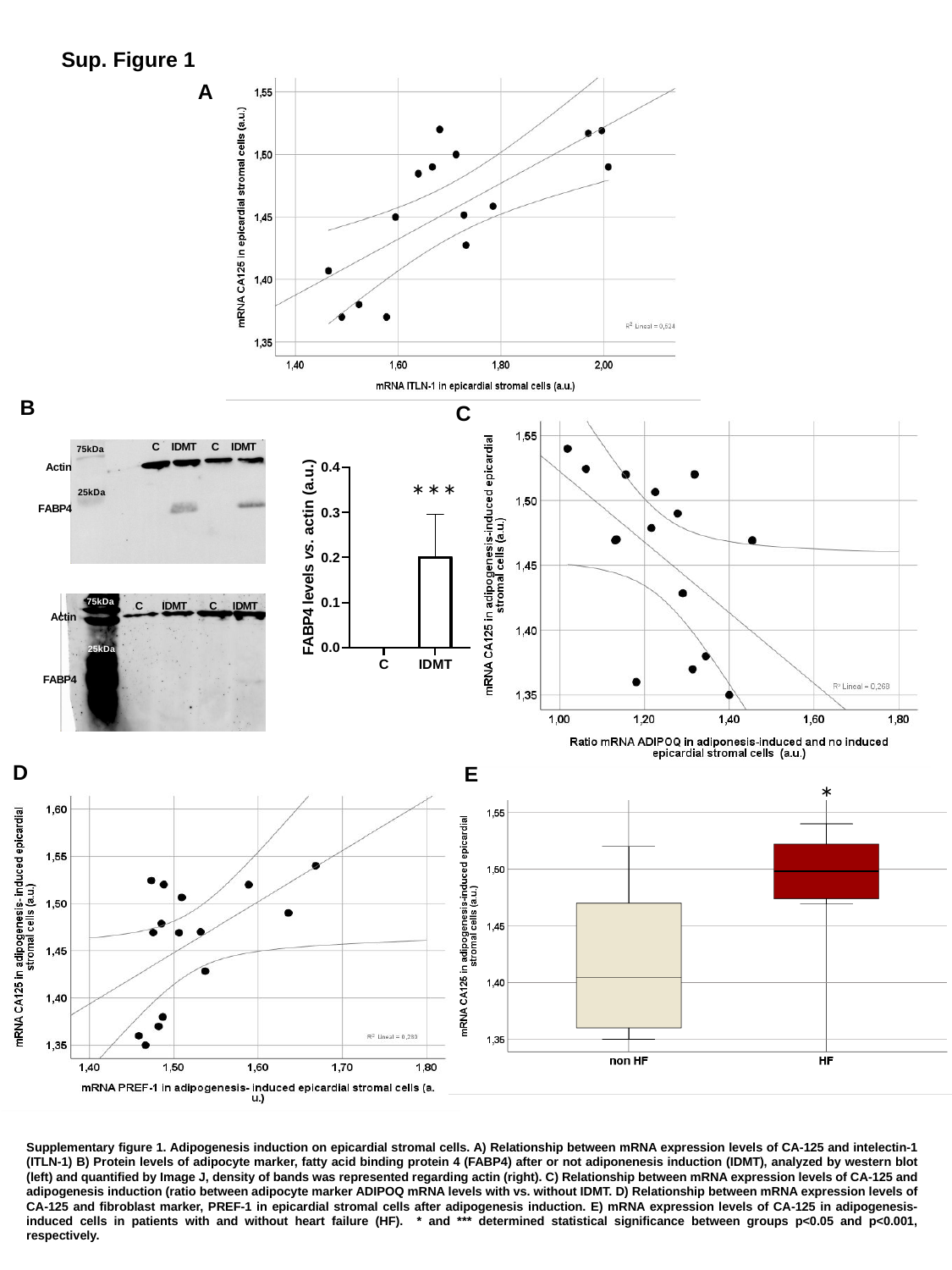

Sup. Figure 1
A
B
C
***
D
E
*
Supplementary figure 1. Adipogenesis induction on epicardial stromal cells. A) Relationship between mRNA expression levels of CA-125 and intelectin-1 (ITLN-1) B) Protein levels of adipocyte marker, fatty acid binding protein 4 (FABP4) after or not adiponenesis induction (IDMT), analyzed by western blot (left) and quantified by Image J, density of bands was represented regarding actin (right). C) Relationship between mRNA expression levels of CA-125 and adipogenesis induction (ratio between adipocyte marker ADIPOQ mRNA levels with vs. without IDMT. D) Relationship between mRNA expression levels of CA-125 and fibroblast marker, PREF-1 in epicardial stromal cells after adipogenesis induction. E) mRNA expression levels of CA-125 in adipogenesis-induced cells in patients with and without heart failure (HF). * and *** determined statistical significance between groups p<0.05 and p<0.001, respectively.
